# Eco-friendly Synthesis of Carbon Quantum Dots as an Effective Adsorbent

**DOI:** 10.1007/s10895-022-03085-z

**Published:** 2022-11-26

**Authors:** Hebat‑Allah S. Tohamy, Mohamed El‑Sakhawy, Samir Kamel

**Affiliations:** Cellulose and Paper Department, National Research Centre, Cairo, 12622 Egypt

**Keywords:** Carbon quantum dots, Graphene quantum dots, Cellulose, Fluorescence, Water treatment, Agricultural wastes

## Abstract

Fluorescent carbon quantum dots (CQDs) were prepared by an economical, green, and single-step procedure with the assistance of microwave heating of urea with bagasse (SCB), cellulose (C), or carboxymethyl cellulose (CMC). The prepared CQDs were characterized using a series of spectroscopic techniques, and they had petite size, intense absorption in the UV, and excitation wavelength-dependent fluorescence. The prepared CQDs were used for Pb(II) adsorption from an aqueous solution. The removal efficiency percentages (R %) were 99.16, 96.36, and 98.48% for QCMC, QC, and QSCB, respectively. The findings validated the efficiency of CQDs synthesized from CMC, cellulose, and SCB as excellent materials for further utilization in the environmental fields of wastewater pollution detection, adsorption, and chemical sensing applications. The kinetics and isotherms studied found that all CQDs isotherms fit well with the Langmuir model than Freundlich and Temkin models. According to R^2^, the pseudo-second-order fits the adsorption of QCMC, while the first-order one fits with QC and QSCB.

## Introduction

Agriculture wastes are the most reliable source for manufacturing valuable products that require fewer chemical reagents (i.e., eco-friendly) to remove metal ions from wastewater. Agriculture trash that builds up harms the environment. As a result, demand for recycling. A more significant attempt has recently been made to utilize agricultural waste. One of them was the creation of carbon nanomaterials using bagasse (SCB) as a source [1].

Carbon quantum dots (CQDs) consist of graphene quantum dots (GQDs) and carbon quantum dots (CQDs) as carbon nanomaterials in the size of 10 nm with a large surface area. GQDs are small graphene (G) sheets that are zero-dimensional (i.e., dimensionless (0-D)). GQDs have oxygen-containing functional groups (e.g., OH, C = O, and C­–O–C). CQDs are ball-shaped nanoparticles (NPs) with sp2 hybrid carbon and graphene oxide sheets (GO) connected by diamond-like sp3 hybrid carbon [2]. CQDs have been prepared by several methods, such as laser ablation of graphite, pyrolytic carbonization, and hydrothermal and solvothermal heating of organic compounds. Several studies have shown that using microwave heating is an appropriate strategy for creating quicker and more affordable processes for CQD synthesis [3, 4]. Typical CQDs reported so far consist of sp^2^ hybrid carbon cores and O- and N-containing functional groups distributed on their whole surface. The intrinsic hydrophobic nature of the carbon cores is shielded by the layer of hydrophilic groups, leading to a stable aqueous dispersion of functionalized CQDs with excellent biocompatibility [5]. Additionally, CQDs are fluorescent dyes with distinctive optical characteristics. Because of their biocompatibility, affordability, and environmental friendliness, CQDs have been successfully used in wastewater treatment [6].

Agricultural wastes and carbohydrates (e.g., cellulose, carboxymethyl cellulose, etc.) have been widely used to produce carbon materials owing to their sustainability [7]. With their high specific surface area, nitrogen-doped CQDs (N-CQDs) can improve the adsorption of metal ions from wastewater. A previous study revealed that the N-CQDs could remove 37% of Cd(II) and 75% of Pb(II) from wastewater through an adsorption process [8].

In the present work, an aqueous sodium hydroxide/ urea solution was used to dissolve the cellulosic materials, followed by microwave heating, giving different carbon quantum dots. Then, the carbon quantum dots were utilized to adsorb Pb(II) from an aqueous solution. The kinetics and isotherms of the adsorption process at different conditions were studied. Also, the fluorescence of carbon quantum dots with/ without Pb(II) was studied.

## Materials and Methods

### Materials

Bagasse (SCB) was provided by Quena Company for Paper Industry Egypt. SCB was air-dried and homogenized to avoid compositional differences between batches and ground to mesh size 450 micron. All chemicals used were of analytical grades and used without further purification.

### Extraction of Cellulose from Bagasse

Based on the raw material, SCB was hydrolyzed with 1.5% HCl using a liquor ratio of 1:10 at 120 °C for two hours. Based on the weight of SCB, 20% NaOH was applied to the hydrolyzed bagasse in a liquid ratio of 1:7 at 170 °C for two hours. The pretreated bagasse's remaining lignin was eliminated using sodium chlorite bleaching. To get rid of the residual traces of lignin and other components, cellulose was mercerized using 17.5% NaOH [9].

### Synthesis of Carboxymethyl Cellulose (CMC)

In a typical procedure, to a suspended bleached pulp of 15 g/400 ml isopropyl alcohol, 30% NaOH solution was added dropwise for 30 min. The mixture was left under stirring for 1 h at room temperature. After that, 18 g of mono chloroacetic acid (MCA) dissolved in isopropyl alcohol was added dropwise to the mixture for 30 min. The mixture was allowed to react under stirring at 55 °C for 3.5 h. Finally, the liquid was drained off, and the product was stirred in 70% methanol, separated by filtration, and dried at 60 °C. The degree of substitution of the carboxyl group in CMC was assessed by potentiometric titration according to the standard method (DS ~ 0.7) [9].

### Preparation of N–doped Graphene Quantum Dots and Carbon Quantum Dots

CQDs were prepared from SCB, CMC, and cellulose by dissolving 0.03 g of these carbon sources in 0.07 g NaOH and 2.4 g urea and left for one night in the freezer separately to form a homogeneous solution. When it reached room temperature, each dissolved mixture was ultra-sonicated for 2 min, followed by microwave treatment at 700 W for 7 min to turn into yellow/brown crude CQDs solid [10].

### Pb(II) Adsorption Study

To characterize the sorption efficiency of the prepared CQDs toward removing Pb(II) from water, a comparative removal efficiency (R %) of CQDs was studied at different contact times, temperatures, and pHs. The CQDs were filtered from the solution after Pb(II) adsorption. R % was calculated using Eq. (1):

1 $$R\,\%=\frac{(C0-Ct)}{C0}\times 100$$

where C_O_ and Ct are the Pb(II) concentrations (mg/l) in solution before and after adsorption. V is the volume of solution (L). M is the amount (g) of the sorbent employed in the adsorption experiment [9, 11].

Pseudo-first-order and Pseudo-second-order can be determined from Eqs. (2) and (3).

2 $$\mathrm{ln}\left[qe-qt\right]=\mathrm{ln}\;qe-K1t$$

3 $$\frac{\mathrm{t}}{\mathrm{qt}}=\frac{1}{\mathrm{K}2\mathrm{qe}2}+\frac{\mathrm{t}}{\mathrm{qe}}$$

where qe and qt are the amounts of Pb(II) adsorbed (mg/g) at equilibrium sorption capacity and time t, respectively. K1 (min^−1^) is the Pseudo-first-order rate constant of adsorption. K2 (g/mg/min) is the rate constant of Pseudo-second-order adsorption. Values of qe2 and K2 were calculated from the slope and intercept of the plot of t/qt against t, respectively [9, 11, 12].

Langmuir, Freundlich, and Temkin's isotherms can be determined from Eqs. (4), (5) and (6), respectively.

4 $$\frac{\mathrm{Ce}}{\mathrm{qe}}=\frac{1}{\mathrm{Kqm}}+\frac{\mathrm{Ce}}{\mathrm{qm}}$$

5 $$\mathrm{log\,qe}=\mathrm{log\,Kf}+\frac{1}{\mathrm{n}}\mathrm{log\,Ce}$$

6 $$qe=\left[Rt/b\right]lnA+\left[Rt/b\right]lnCe$$

where qm (mg/g) is the maximum removal capacity, Kf is the adsorption capacity. RT/b = *B* (J/mol) which is Temkin constants related to the heat of sorption, *A* (L/g) is the equilibrium binding constants corresponding to the maximum binding energy, R is the universal gas constant, and T (K) is the absolute temperature [9, 12].

Thermodynamic parameters can be determined from Eqs. (7), (8) and (9):

7 $$\mathrm{ln\,Kd}=\frac{\Delta \mathrm{S}}{\mathrm{R}}-\frac{\Delta \mathrm{H}}{\mathrm{RT}}$$

8 $${\mathrm{K}}_{\mathrm{d}}=\frac{\mathrm{Ci}-\mathrm{Ce}}{\mathrm{Ce}}\times \frac{\mathrm{v}}{\mathrm{m}}$$

9 $$\Delta \mathrm{G}=-{\mathrm{RT\,ln\,K}}_{\mathrm{d}}$$

where T is the temperature in Kelvin, R is the universal gas constant, and Kd is the distribution coefficient. ΔH and ΔS can be obtained from the slope and intercept of Van't Hoff plot of ln K versus 1/T, respectively [9, 11, 12].

### Characterization

FTIR spectra are recorded using an FTIR spectrophotometer (Nexus 670, Nicolet, USA) in the 4000–400 cm^−1^ with a spectra resolution of 4.0 cm^−1^. Furthermore, transmission electron microscope (TEM) images were taken with a JEOL JEM-2100 electron microscopy at an acceleration voltage of 120 kV. Atomic absorption PerkinElmer 3110, USA, was used to quantify the metal ions. The UV–vis absorption spectrum was recorded by a UV–Vis spectrophotometer (JASCO V-630, Japan) using a 1-cm path length quartz cell. The quantum yield was calculated according to Eq. (10):

10 $$\mathrm{Qx}=\mathrm{Qst}.\frac{mx}{mst.}{(\frac{\eta \mathrm{x}}{\eta \mathrm{st}.})}^{2}$$

where "Q" is the quantum yield, "m" is the slope from the plot of fluorescence vs absorbance, "ɳ" is the refractive index of the solvent, the "x" indicates the unknown sample, and "st." refers to methylene blue standard solution in water (0.1 M) [13].

## Results and Discussion

### FTIR Spectra

**Fig. 1:**
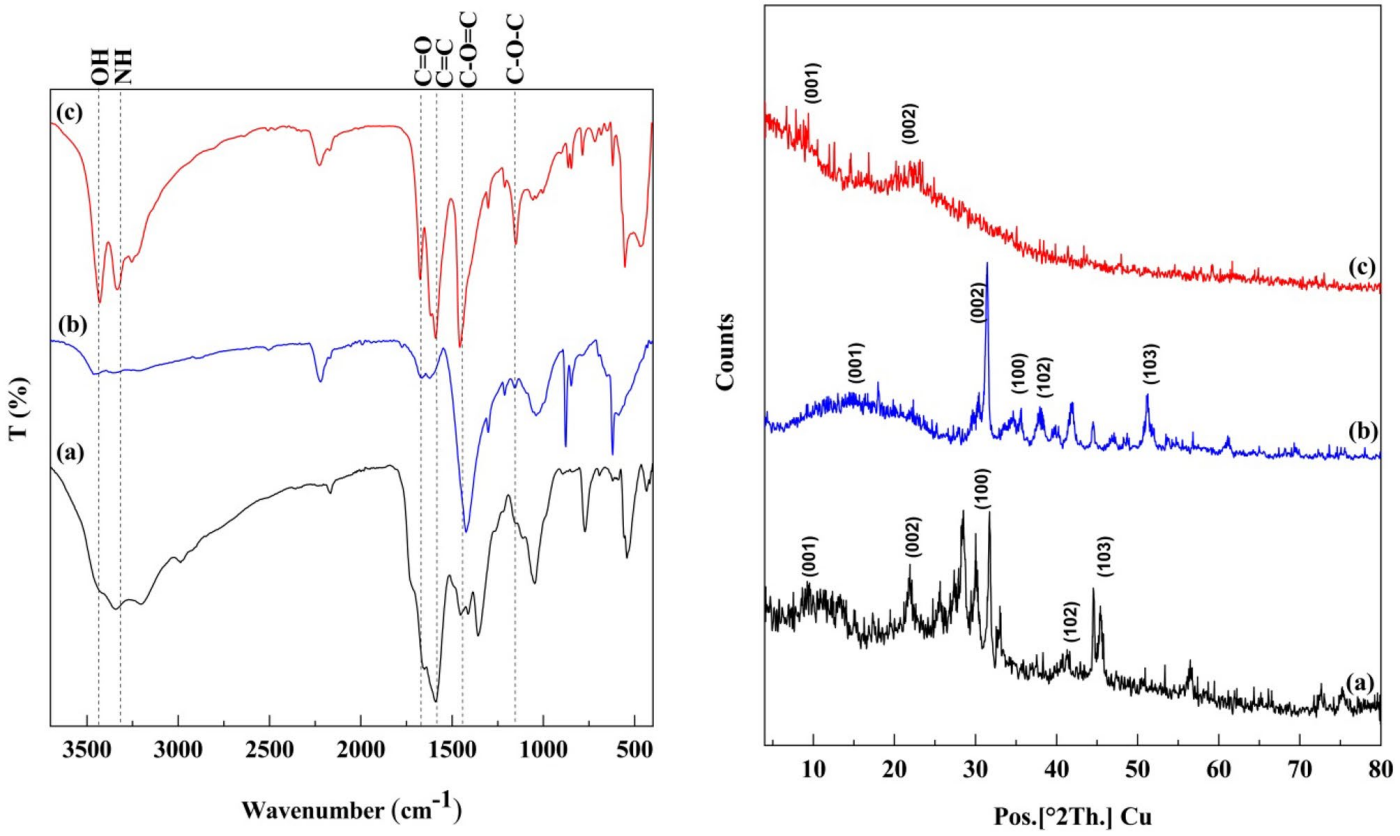
The FT-IR spectra and X–ray pattern of **a** QCMC, **b** QC, and **c** QSCB

The IR spectra of the prepared carbon quantum dots show absorption bands at 1590–1621 and 1654–1675 cm^−1^ assigned to the fingerprints of amide II and amide I bands, respectively (Fig. 1) [3]. A shift in the amide bands of the three CQDs is mainly due to the difference in the chemical structure of the starting materials. The microwave heating procedure causes pyrolysis of the reactants, resulting in CQDs containing amide groups. The bands at 3344–3463, 1590–1621, and 1423–1590 cm^−1^ are attributed to O–H/N–H, C = O, and C = C groups, respectively [14]. The bands centered at 1212–1457 and 1158–1358 cm^−1^ are usually attributed to C–O = C and C–O–C stretching Vibrations [15–17]. Functional groups resulting from urea, such as N–H and C–N, are centered at 3203–3359 and 1040–1150 cm^−1^, respectively [12]. In addition, the FTIR spectrum revealed the presence of hydrophilic surface functional groups, such as OH and NH groups, over the CQDs surface, thus imparting excellent water solubility and stability [9].

### X–ray Analysis

The XRD pattern revealed the known peaks of GO with peaks at 2θ = 11.1, 14.3, and 9.0° related to the (001) plane, with the interlayer d spacing of 0.04, 0.31, and .098 nm and at 2θ ~ 21.8, 31.4 and 22.8° related to (002) plane due to the presence of GO for QCMC, QC, and QSCB, respectively (Fig. 1) [5]. The increase in the d value indicates an amorphous nature attributed to introducing of more oxygen and nitrogen-containing groups in the case of QC [18].

The XRD spectrum in Fig. 1 confirms that the synthesized QC and QCMC are crystalline. The peaks at 31.4, 38.2, 41.7, and 51.2° for QC and 27.8, 31.3, 41.8, and 44.6° for QCMC correspond to the (002), (100), (102), and (103) crystal planes in which (002), (100), and (102) represent graphite (sp^2^) and (103) represents diamond (sp^3^) like carbon [19]. The QCMC has a broad diffraction peak at 2θ = 27.8 and another at 2θ = 44.6°, indicating that the CMC is partly crystalline [9]. Respectively, the CrI % was 54.11, 33.3, and 44.53% for QCMC, QC, and QSCB.

### TEM Analysis

**Fig. 2:**
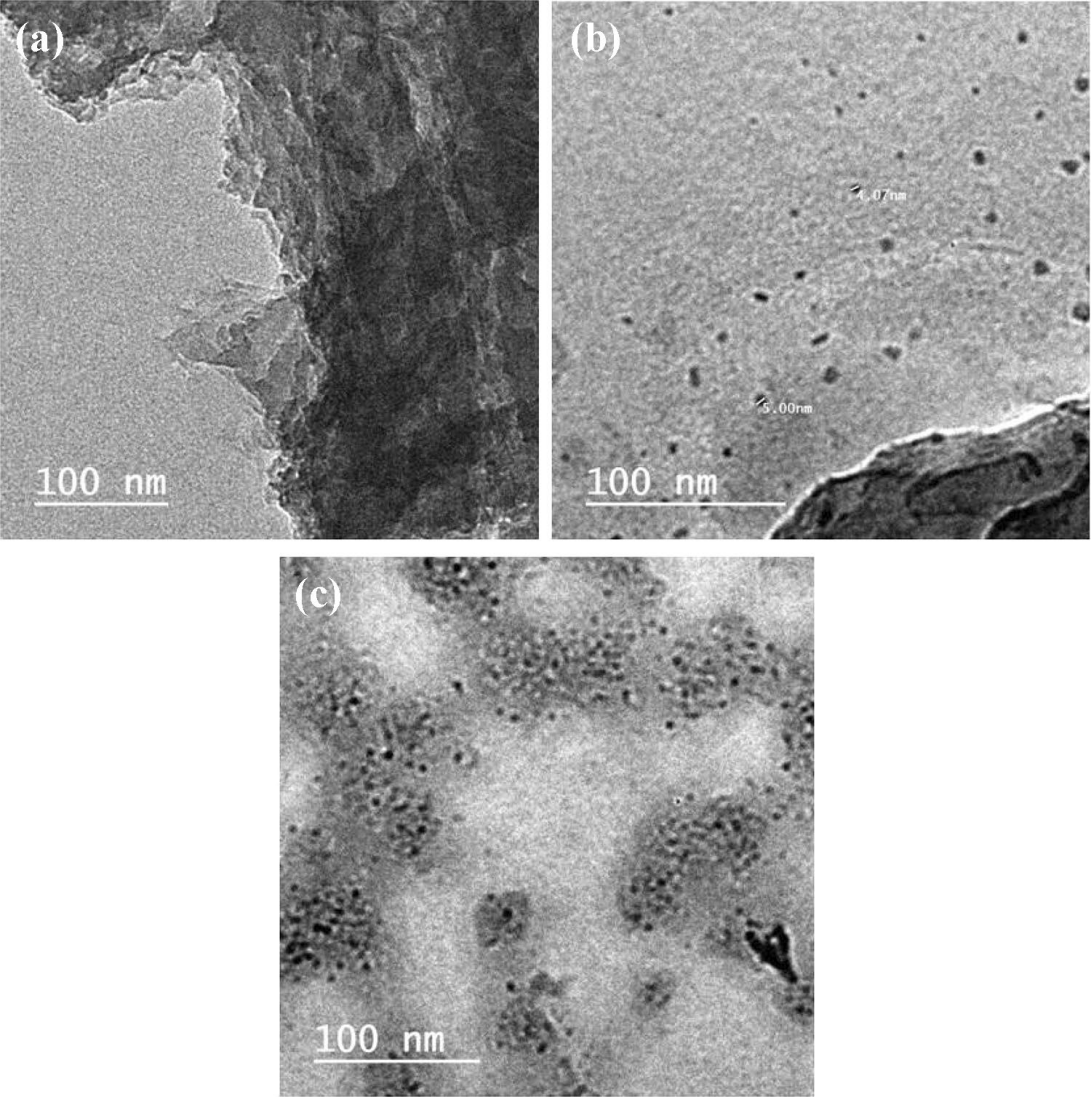
TEM images of **a** QCMC, **b** QC, and **c** QSCB

TEM images (Fig. 2) reveal that QCMC is graphene oxide in sheet-like nature. The color gradient on the sheet's edges is proof that GO consists of a few layers of sheets that stack together, while QC and QSCB are CQDs [5, 7, 9]. The CQDs were spherical and uniform in size 5.00 and 2.35 nm for QC and QSCB, respectively, indicating mono-dispersity. QC contains CQDs embedded GO sheets, while QSCB is pure C-QDs.

### UV Spectra

**Fig. 3:**
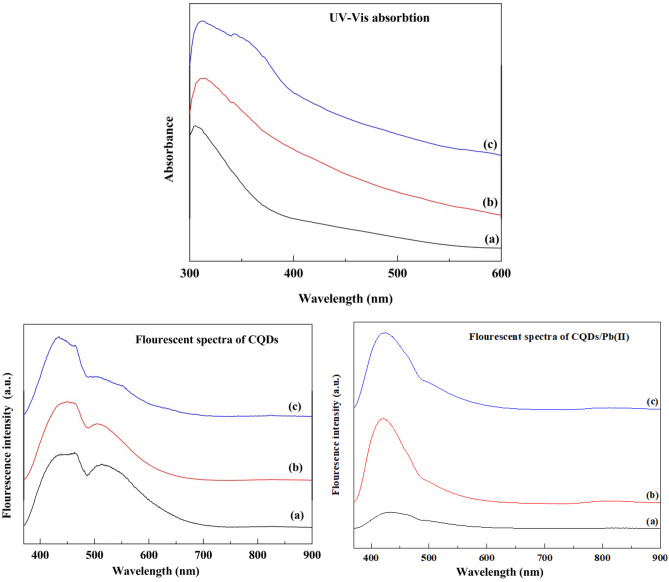
UV–vis absorption spectrum, fluorescent spectra without Pb(II), and fluorescent spectra without Pb(II) of **a** QCMC, **b** QC, and **c** QSCB

The UV–vis spectrum of CQDs in Fig. 3 shows typical optical absorption in the UV region, with a tail extending to the visible range. The spectrum has two main absorption features: an intensive peak at 310, 310, and 306 nm due to the π–π* transition of C = C bonds at QCMC, QC, and QSCB, respectively. A shoulder peak at 344 and 342 nm was assigned to the n– π* transition of C = O bonds for QCMC and QC [10]. The increase of QY for QC may be mechanistically related to its higher doping of nitrogen (2.03) compared with those of QCMC (0.38) and QSCB (0.33). We conclude that the high QY in QC is most likely due to the charge transfer caused by nitrogen atoms [16].

### Fluorescent Spectra

The fluorescence emission spectra of CQDs are displayed in Fig. 3. All CQDs were excited at 350 nm and showed the maximum emission wavelength of 461.0, 456.5, and 440 nm for QCMC, QC, and QSCB, respectively. Fluorescence emission is caused by C = O/C = N moieties of the CQDs' surfaces [20, 21]. The emission peaks at 513, 505, and 506 nm are derived from the oxygen vacancy defects in QCMC, QC, and QSCB, respectively, as mentioned by Dong et al. [22]. This difference in the position of the peaks is attributed to the variation in CQD size [18]. The quality of the CQDs obtained is associated with the full width of half maximum (FWHM), calculated as 84.5, 77.0, and 88.0 nm, similar to other previously reported CQDs [21].

The maximum emission wavelength of QCMC, QC, and QSCB after Pb(II) adsorption were 432.4, 419.7, and 423.4 nm, respectively. This emission could theoretically prove the use of these materials as sensors. Furthermore, the oxygen vacancy defects in the case of QCMC/ Pb(II), QC/ Pb(II), and QSCB/ Pb(II) are almost hidden and may be due to Pb(II) adsorption. The changes in fluorescence intensity (ΔF) of QCMC, QC, QSCB, QCMC/ Pb(II), QC/ Pb(II), and QSCB/ Pb(II) are shown in Fig. 3. Compared with QCMC, QC, and QSCB upon the addition of Pb(II), the ΔF of QCMC/ Pb(II), QC/ Pb(II), and QSCB/ Pb(II) is increased upon the addition of Pb(II) at an excitation wavelength of 350 nm. This categorically proves that the as-prepared QCMC, QC, and QSCB are ideal candidates for sensitive detection of Pb(II). These findings validated the efficiency of CQDs synthesized from CMC, cellulose, and SCB as excellent materials for further utilization in chemical sensing applications.

### Thermogravimetric Analysis (TGA/ DTG)

**Fig. 4:**
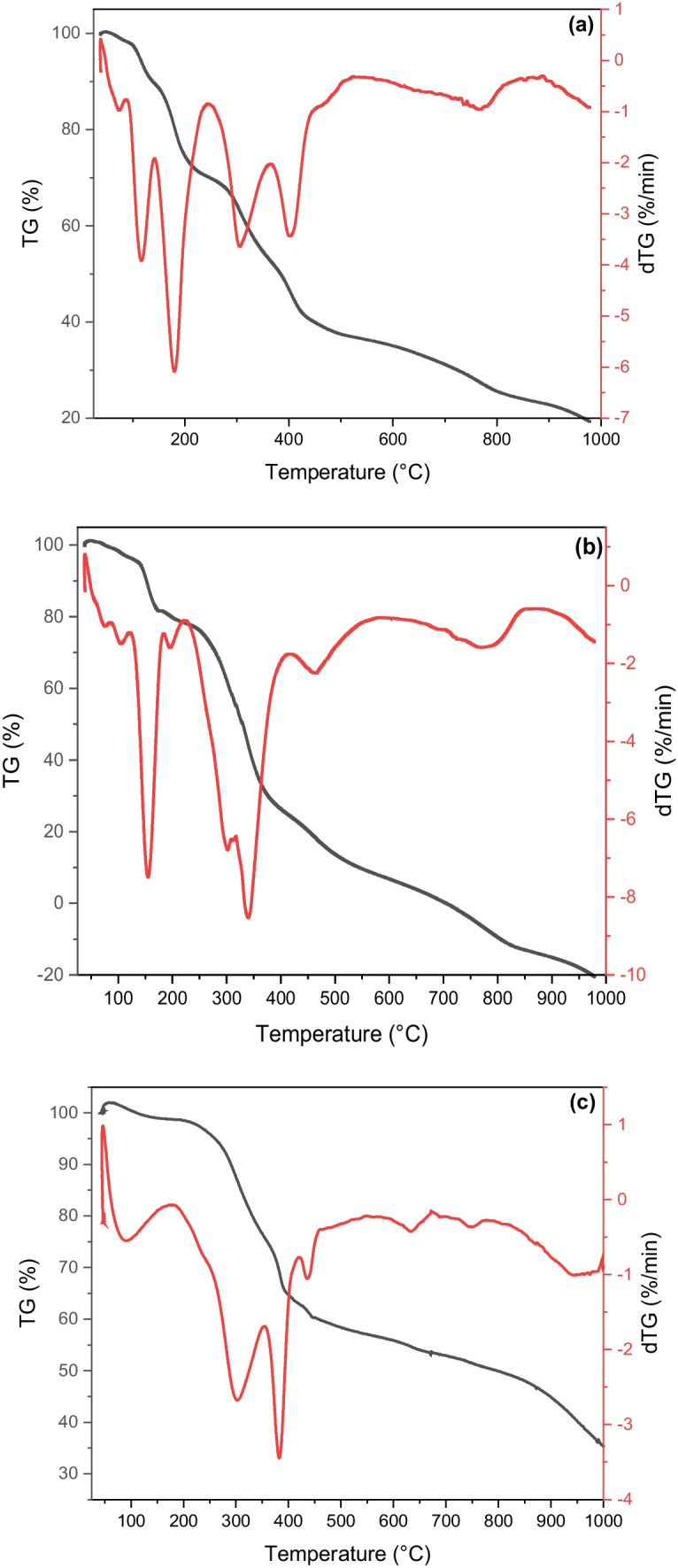
TGA/DTG of **a** QCMC, **b** QC, and **c** QSCB

Thermogravimetry (TG) and its derivative (DTG) is an analytical technique used to determine the thermal stability and the fraction of volatile components by monitoring the change of weight that occurs with the heating of the sample. Figure 4 and Table 1 represent the thermogravimetric analysis of the prepared CQDs. It is known that a sudden temperature change generates a thermal shock, and functionalities are taken out from QCMC, QC, and QSCB lattices as water vapor, CO, and CO_2_. The TGA/DTG of QCMC, QC, and QSCB showed a weight loss of 63.80, 97.13, and 64.67%, respectively, at 100 °C, which indicated the presence of a fraction of non-volatile components [9, 12].

**Table 1:** TGA/ DTG data of QCMC, QC, and QSCB

**Sample**	**Stage**	**TGA range, °C**	**DTA peak, °C**	**Mass loss** **(%)**	**N**	**R**^**2**^	**A (s**^**−1**^**)**	$\Delta \mathbf{H}$ **(Kj mol**^**−1**^**)**	$\Delta {\varvec{s}}$ **(Kj mol**^**−1**^**)**	$\Delta {\varvec{G}}$ **(Kj mol**^**−1**^**)**	**SE**	**E,** **(kJ mol**^**−1**^**)**
**QCMC**	**1st**	39.20–86.42	68.85	3.02	0.5	0.9998	3.02	2.78	–0.23	83.76	57X10^−4^	5.62
	**2nd**	91.09–241.64	112.83 176.76	9.28	2.5	0.9999	4.20	–3.20 –3.73	–0.23 –0.23	87.51 102.59	94X10^−4^	4.66
	**3rd**	257.88–453.84	303.17 403.65	18.82	2	0.9998	2.87	1.19	–0.23 –0.24	139.19 164.16	88X10^−4^	5.98
	**4**^**th**^	508.45–977.70	764.60	32.70 **∑ML % = 63.82** **∑RW % = 36.18**	1	0.9959	2.97	7.56	–0.24	263.08	90X10^−3^	16.18 **∑E = 32.44**
**QC**	**1st**	39.25–192.21	151.45	5.14	1.5	0.9695	12.27	–3.52	–0.22	92.83	30X10^−2^	9.49
	**2nd**	231.43–406.02	297.42 & 336.92	17.05	0	0.9988	2.92	1.25 0.93	–0.24 –0.24	138.96 148.51	29X10^−3^	6.00
	**3rd**	418.93–569.07	466.69	53.16	2	0.9997	3.63	11.87	–0.24	190.79	30X10^−3^	18.03
	**4th**	609.79–969.82	779.26	18.94 **∑ML % = 91.29** **∑RW % = 8.71**	1.5	0.9990	3.36	12.62	–0.24	270.95	70X10^−3^	21.38 **∑E = 54.90**
**QSCB**	**1st**	49.21–166.51	83.14	1.29	1	0.8538	2.06	15.00	–0.29	121.06	40X10^−2^	17..96
	**2nd**	191.70–414.18	299.43 & 380.31	25.63	3	0.8515	3.71	35.19 –5.43	–0.23 –0.24	172.26 151.72	34X10^−1^	39.95
	**3rd**	415.98–468.19	435.68	12.59	0.5	0.9979	2.87	4.37	–0.24	176.84	57X10^−3^	10.26
	**4th**	576.80–668.28	631.34	24.22 **∑ML % = 63.73** **∑RW % = 36.27**	0.5	0.9954	2.97	8.90	–0.24	230.57	10X10^−2^	16.42 **∑E = 84.59**

The thermal decomposition processes of QCMC, QC, and QSCB could be divided into four major reaction steps, where the first weight loss was between 39.20—86.42, 39.25—192.21, and 49.21—166.51 °C, with a maximum at 68.85, 151.45, and 83.14 °C with mass loss (ML %) of 3.02, 5.14, and 1.29%, respectively, was likely caused by the loss of moisture content. The second splitter was between 91.09—241.64, 231.43—406.02, and 191.70—414.18 °C; with a maximum at 112.83/ 176.76, 297.42/ 336.92, and 299.43/ 380.31 °C. Due to dehydroxylation and pyrolytic fragmentation, the MLs were 9.28, 17.05, and 25.63% for QCMC, QC, and QSCB.

The third decomposition step was between 257.88—453.84, 418.93—569.07, and 415.98—468.19 °C, with a maximum of 303.17/ 403.65, 466.69, and 435.68 °C. The MLs were 18.82, 53.16, and 12.59% for QCMC, QC, and QSCB, ascribed to the decomposition of the carbonaceous residues to form low molecular weight gaseous products.

The fourth decomposition step was between 508.45—977.70, 609.79—969.82, and 576.80—668.28 °C; with a maximum at 764.60, 779.26, and 631.34 °C The MLs were 32.70, 18.94, and 12.31% for QCMC, QC, and QSCB, which was ascribed to the decomposition of The third and fourth steps are ascribed to the depolymerizations and carbonization to form low molecular weight gaseous products [9, 11, 12]. The appearance of small peaks at temperature ranges from 700 to 900 °C for QSCB probably represents char oxidation.

The onset temperature, DTG peaks, and activation energies (Ea) of the volatilization steps of QCMC (the 2nd, 3rd, and 4th degradation steps) are lower than the volatilization steps of QSCB and QC (the 2nd, 3rd, and 4th degradation steps) (Table 1). Increasing in Ea of QSCB indicates thermal stabilization of the conjugate structure. Therefore, it requires high Ea to degrade [9, 12].

### Pb(II) Adsorption Study

**Fig. 5:**
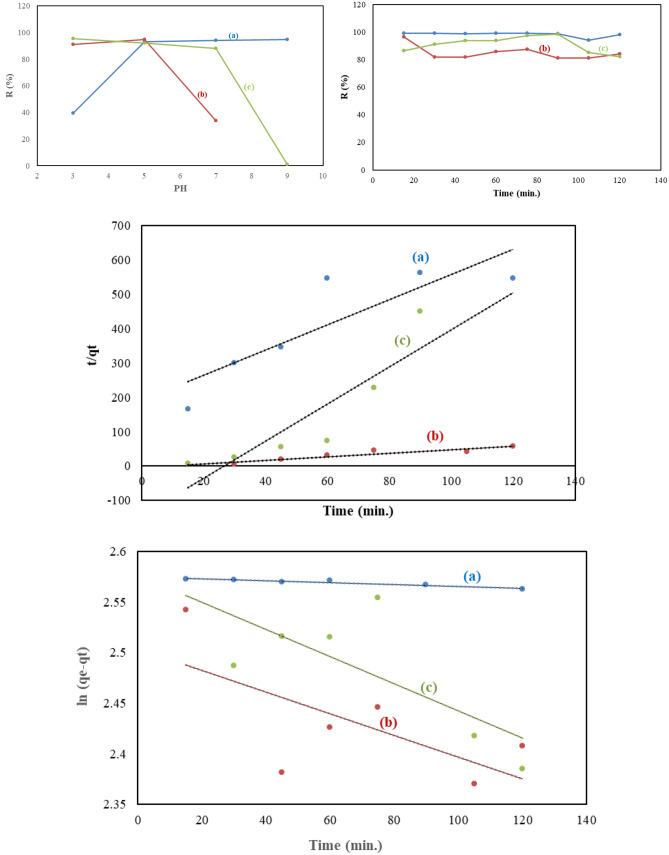

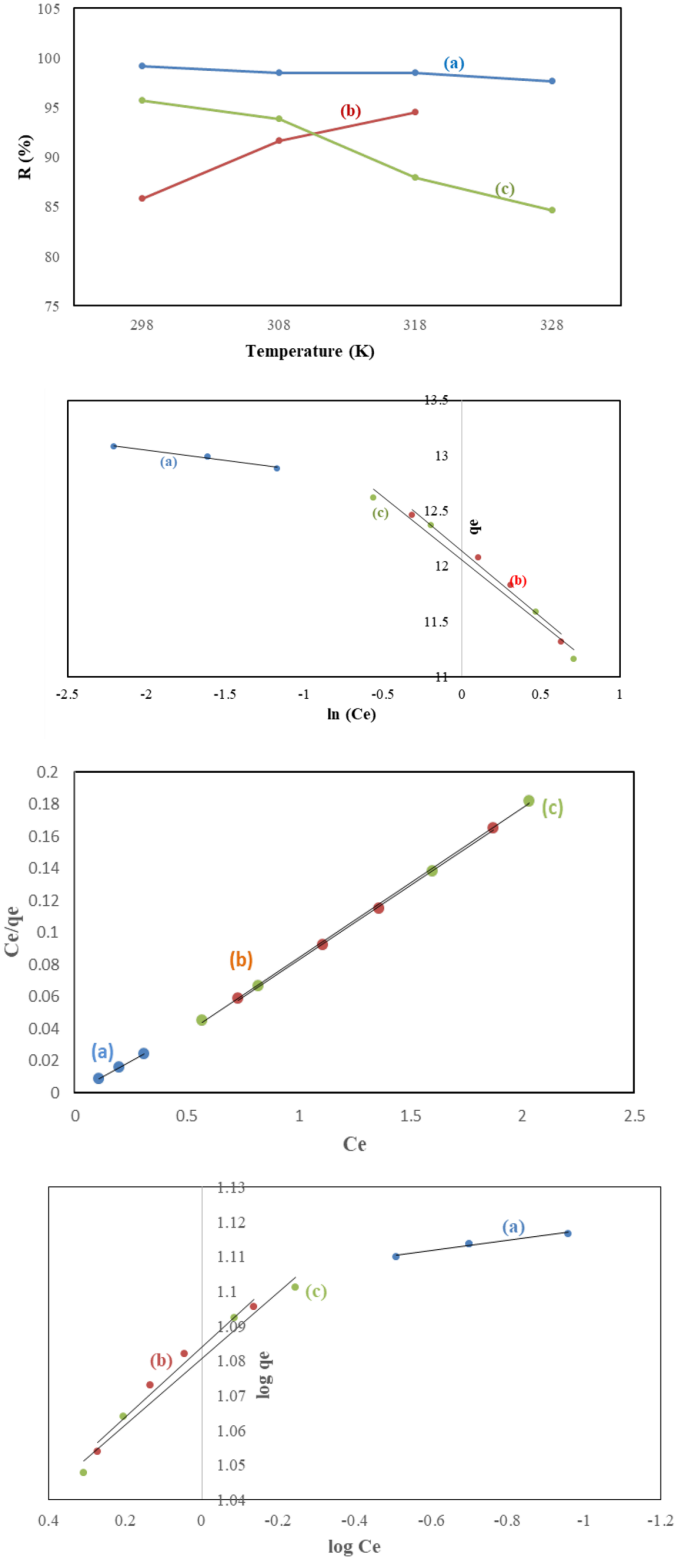
Effect of contact time, pH, and temperature on the adsorption of Pb(II) onto QCMC, QC, and QSCB and their kinetic models

The prepared CQDs were examined to remove Pb(II). The effect of contact time on adsorption efficiency was studied at different times, namely 15, 30, 45, 60, 75, 90, 105, and 120 min. As shown in Fig. 5a, the affinity of CQDs towards Pb(II) was not the same, and the removal of Pb(II) by CQDs adsorbents was found to be fast at first due to the presence of more free functional groups [12, 23]. Then, the removal rate became slow, and there was no remarkable increase in the adsorption rate observed after 75, 15, and 90 min for QCMC, QC, and QSCB, respectively. So, the optimum time for the adsorption of Pb(II) on QCMC, QC, and QSCB at 25 °C are 75, 15, and 90 min, respectively. Pb(II) adsorption to QCMC was much higher than other CQDs (R% = 99.16) and stable until 75 min. This may be due to the presence of high oxygen (carboxymethyl content) in the starting material (CMC) as compared to cellulose and SCB (R% = 96.36 and 98.48). After 75, 15, and 90 min for QCMC, QC, and QSCB, respectively, the adsorption rate decreased due to the leaching process, which proves the difference in the morphology of the prepared CQDs [9, 12]. Our results match that of Yahaya Pudza et al. which implied that CQDs are highly adsorbents for effective Pb(II) adsorption with R% = 80.6% [6].

The pseudo-first-order and pseudo-second-order equations are utilized to model the kinetics of Pb(II) adsorption onto CQDs. Concerning the values of R^2^ presented in Table 2, it is seen that the pseudo-second-order model gave a better fit to the adsorption data of QCMC (i.e., chemisorptions). In comparison, the first-order one better fits the adsorption data of QC and QSCB (physisorption) [9]. The values obtained in the Pseudo-first-order are still suitable for describing the kinetics of Pb(II) sorption in the case of QC and QSCB. These values elucidate the surface processes involving chemisorption and physisorption in the adsorption of Pb(II) by QC and QSCB [11, 12].

**Table 2:** Comparison between the estimated adsorption rate constants, rate constants, and correlation coefficients associated with the pseudo-first-order, the pseudo-second-order rate, Langmuir, Freundlich, and Temkin models parameters for adsorption of Pb(II) onto the prepared QDs

**Kinetic model**	**Parameter**	**Adsorbent**		
		**QCMC**	**QC**	**QSCB**
**Pseudo first order**	**q**_**exp.**_	13.08	12.71	12.99
	**q**_**calc.**_	13.21	10.07	12.33
	**K**_**1**_	30X10^−4^	43X10^−4^	41X10^−4^
	**R**^**2**^	0.901	0.447	0.517
**Pseudo second order**	**q**_**calc.**_	48X10^−1^	3.22	0.71
	**K**_**2**_	78X10^−3^	28X10^−2^	61X10^−2^
	**R**^**2**^	0.752	0.864	0.785
**Langmuir**	**q**_**m**_ **R**^**2**^	12.77 0.999	10.67 0.999	10.70 0.999
**Freundlich**	**K**_**f**_ **1/n** **R**^**2**^	3.01 0.01 0.967	2.96 0.10 0.972	2.95 0.09 0.972
**Kinetic isotherms**	**ΔS (kJ/mol)**	–0.05	0.06	–0.09
	**ΔH (kJ/mol)**	–25X10^3^	13X10^3^	–33X10^−3^
	**ΔG (kJ/mol)**			
**Temkin isotherms**	**298 K** **308 K** **318 K** **328 K** **A (L/g)** **B (J/mol)** **R**^**2**^	–11.84 –10.69 –11.03 –10.16 1.34X10^28^ –5.11 0.968	–4.46 –6.11 –7.50 –5.89 19X10^3^ –0.81 0.976	–6.7286 –7.9395 –5.233 –4.65 31X10^3^ –0.86 0.980

The effect of temperature on the adsorption capacity of Pb(II) on CQDs was performed from 298 to 328 K for 60 min. As shown in Fig. 5d, with the increase in temperature from 298 to 328 K, the removal of Pb(II) by QCMC and QSCB decreases, i.e., an exothermic process. This is due to the thickness of the boundary layer decreasing at high temperatures and the tendency of Pb(II) to escape from the QCMC, and QSCB surfaces to the solution phase increased. On the other hand, Pb(II) removal by QC increased with increasing temperature, i.e., an endothermic process due to the nature of QC. The endothermic process is due to the pore size enlargement and activation of the QC surface. The difference in the adsorption attitude of Pb(II) proves that the samples are differ in morphology [9, 11, 12]. The higher K_f_ of QCMC indicates that the QCMC is more reactive [11, 12]. 1/n is a function of the strength of adsorption. This study 1/n < 1 indicates normal adsorption [24]. All CQDs isotherms fit well with the Langmuir model than Freundlich and Temkin models, since R^2^ was high (Fig. 5e, f and Table 2). So, it can be derived that the surfaces of CQDs are homogeneous, and surface adsorption is a monolayer. The positive value of ΔH for QCMC and QSCB is due to the endothermic process, while the negative value for QC is due to the exothermic process. The sorption process is spontaneous due to the negative values of ΔG. The ΔS changes are positive for QC; it means that the increased randomness appeared on the solution interface during the exchange of Pb(II) (Table 2) [9, 11, 12].

In addition, the pH value of the Pb(II) solution affects the removal efficiency; therefore, the effect of pH was studied over the pH range of 3–9 (Fig. 5h). With increasing pH from 3 to 5, the removal of Pb(II) was increased. The pH can influence the removal of Pb(II) by changing the surface charges of QCMC, QC, and QSCB. At acidic conditions (pH ≈ 3–5), QCMC, QC, and QSCB could remove Pb(II) at minimal adsorption capacity and increase with pH. The optimum adsorption was obtained at pH 9, 5, and 3 for QCMC, QC, and QSCB. This can be explained in acidic conditions where the competition between Pb(II) and H_3_O^+^ for the active sites on QCMC, QC, and QSCB results in a low adsorption uptake. Thus, as the pH increased (alkaline conditions), H_3_O^+^ numbers decreased, which reduced the competition between Pb(II) and H_3_O^+^ for the active sites; hence the adsorption efficiency increased [23, 25]. At alkaline conditions, the adsorption capacities of Pb(II) were higher due to not only the ion exchange of Pb(II) onto QCMC, QC, and QSCB but also due to the formation and precipitation of Pb (OH)_2_ at higher pH values [26, 27]. This condition is undesirable as the lead precipitation could lead to incorrect values of the adsorption uptake [26]. Subsequently, solubility product constants (Ksp) and known concentrations (20 mg/l) of Pb(II) solutions were used to calculate the maximum pH at which these Pb(II) will not occur as hydrolyzed species. The Ksp used for Pb(OH)_2_ was 43 X 10^–20^ [27]. Pb (II) calculated maximum pH values for Pb(II) onto QCMC, QC, and QSCB were 9, 7, and 3, respectively. Therefore, Pb(II) started to precipitate above pH ~ 7 and 3 for QC and QSCB, respectively, and the QC and QSCB were responsible for Pb(II) adsorption when the pH was less than 7 and 3. However, no Pb(II) precipitation occurred for QCMC under pH values ≈ 9 [26–28].

## Conclusions

In conclusion, we developed a facile and rapid approach for the preparing modified Carbon quantum dots (CQDs) by microwave treatment. CQDs have been successfully synthesized by microwave treatment of SCB, CMC, and cellulose to evaluate the effect of the purity of the carbon source on the CQDs' efficiency. QCMC is GO in sheet-like nature. At the same time, QC contains CQDs embedded GO sheets, while QSCB is pure C-QDs. We conclude that the charge transfer caused by nitrogen atoms is most likely the reason for the high QY in QC. Moreover, Pb(II) adsorption to QCMC was much higher than other types of CQDs (R% = 99.16) and stable until 75 min. This can be because of the presence of high oxygen (carboxymethyl content) in the starting material (i.e., CMC) as compared to cellulose and SCB (R% = 96.36 and 98.48). While the removal efficiencies of QSCB and QC are still high. The excellent adsorption, preferable biocompatibility, and nano-scale structure make them prepared CQDs promising for Pb(II) adsorption.

## Data Availability

All data are available within the text.
